# Comparison of the sensitivity of the UKCAT and A Levels to sociodemographic characteristics: a national study

**DOI:** 10.1186/1472-6920-14-7

**Published:** 2014-01-08

**Authors:** Paul A Tiffin, John C McLachlan, Lisa Webster, Sandra Nicholson

**Affiliations:** 1 School for Medicine, Pharmacy and Health, the Wolfson Research Institute, Durham University Queen’s Campus, University Boulevard, Stockton-on-Tees TS17 6BH, UK; 2 School for Medicine, Pharmacy and Health, Durham University Queen’s Campus, University Boulevard, Stockton-on-Tees TS17 6BH, UK; 3 Institute Health Science Education, Barts and The London School of Medicine and Dentistry, Queen Mary University of London, London, UK

**Keywords:** Medical student selection, Educational attainment, Aptitude tests, UKCAT, Socio-economic factors

## Abstract

**Background:**

The UK Clinical Aptitude Test (UKCAT) was introduced to facilitate widening participation in medical and dental education in the UK by providing universities with a continuous variable to aid selection; one that might be less sensitive to the sociodemographic background of candidates compared to traditional measures of educational attainment. Initial research suggested that males, candidates from more advantaged socioeconomic backgrounds and those who attended independent or grammar schools performed better on the test. The introduction of the A* grade at A level permits more detailed analysis of the relationship between UKCAT scores, secondary educational attainment and sociodemographic variables. Thus, our aim was to further assess whether the UKCAT is likely to add incremental value over A level (predicted or actual) attainment in the selection process.

**Methods:**

Data relating to UKCAT and A level performance from 8,180 candidates applying to medicine in 2009 who had complete information relating to six key sociodemographic variables were analysed. A series of regression analyses were conducted in order to evaluate the ability of sociodemographic status to predict performance on two outcome measures: A level ‘best of three’ tariff score; and the UKCAT scores.

**Results:**

In this sample A level attainment was independently and positively predicted by four sociodemographic variables (independent/grammar schooling, White ethnicity, age and professional social class background). These variables also independently and positively predicted UKCAT scores. There was a suggestion that UKCAT scores were less sensitive to educational background compared to A level attainment. In contrast to A level attainment, UKCAT score was independently and positively predicted by having English as a first language and male sex.

**Conclusions:**

Our findings are consistent with a previous report; most of the sociodemographic factors that predict A level attainment also predict UKCAT performance. However, compared to A levels, males and those speaking English as a first language perform better on UKCAT. Our findings suggest that UKCAT scores may be more influenced by sex and less sensitive to school type compared to A levels. These factors must be considered by institutions utilising the UKCAT as a component of the medical and dental school selection process.

## Background

Internationally, demand for access to medical and dental training is high. Aptitude testing has been introduced as an element of the student selection process. Such testing is intended to facilitate the filtering of candidates and also to improve the probability of recruiting individuals who are more likely to succeed as both undergraduates and practising clinicians [1]. The use of tests of intellectual ability, such as the Medical College Admission Test (MCAT), is common in the United States and Canada [2]. Australia similarly uses assessments, such as the Graduate Medical School Admissions Test (GAMSAT), to assess a candidate’s capacity for reasoning and ability at written communication [3]. In the UK the GAMSAT is used by a number of universities as part of the selection process for graduate entry to medical and dental programmes. In contrast, the UKCAT is widely utilised for selection to both undergraduate and graduate entry courses by the majority of UK-based medical schools and roughly half of all dental schools. The test scores are used independently by universities as a component of their admissions process, along with other factors, such as predicted and actual academic achievement. The scores can thus be used either to screen candidates for interview, or to contribute to an overall aggregated holistic assessment score which is then used to decide whether to offer a place for study.

The different usage style of the UKCAT scores by participating medical and dental schools has previously been characterised and reported [4]. How test scores are used within the selection process has previously been reported to be associated with decreased disadvantage for certain under-represented groups (such as those from a state schooled educational background) applying to medical school [5]. In turn this may be translated into higher proportions of such candidates entering UK medical schools that place greater weight on the UKCAT scores as a component of the admissions process. Indeed, medical schools utilising the UKCAT score as a threshold or as a weighted factor (rather than only in ‘borderline’ cases) are more likely to have entrants who are male and from a non-professional social background. A non-significant trend was also reported for entrants to medical schools using the UKCAT as a threshold or factor in the admissions process to be more likely to have attended a state (non-grammar) school compared to those entering institutions placing less emphasis on the test scores. However, it could be argued that how the UKCAT is used by medical and dental schools may be an ‘instrumental variable’ (i.e. a marker of an attitude towards widening participation [WP] issues) rather than a causal factor [5]. If the UKCAT scores were more weakly associated with sociodemographic factors than traditional measures of academic attainment, then this would be evidence that use of the UKCAT in the selection process is a causal factor in producing differences in medical and dental student populations between institutions.

This potential to reduce disadvantage for certain groups of medical and dental applicants is a crucial issue related to UKCAT use and development; in the UK a key aspiration behind the introduction of the test was that it might facilitate WP in these professions. In the UK, issues concerned with WP in professions such as medicine and dentistry largely reflect disparity of socio-economic background [6]. As such it would be desirable to have a test where scores were less influenced by sociodemographic variables such as ethnicity, type of schooling and socioeconomic background. If UKCAT scores were indeed less biased compared to traditional factors used in selection (e.g. A level grades and personal statements) then this would be evidence to justify greater weight being placed on the test results during admissions. In this regard individual UKCAT items have previously been shown to be generally free from differential item functioning (DIF) in terms of *item bias* according to age, ethnicity, sex and social class. Item bias is said to be present when the response (e.g. correct/incorrect) to a test question is partly determined by characteristics other than the trait or ability the instrument is designed to evaluate (i.e. it represents the bias in responses after controlling for ability). Nevertheless, a small number of items may be moderately sensitive to age and ethnicity [7]. However, although DIF is a term sometimes used interchangeably with *item bias*, historically the concept also encapsulates the concept of *item impact*. Item impact can lead to DIF because true differences exist in the level of trait or ability being measured [8]. Therefore, it is still possible that UKCAT scores are sensitive to WP group membership despite relatively little evidence of item bias. Analysis of UKCAT test scores from a cohort of medical entrants to a single university suggested that the UKCAT scores were not significantly associated with school type attended (although the study may have been under powered to detect such an effect) and had some ability to predict performance in knowledge-based medical undergraduate exams [9]. In contrast the scores given to personal statements were significantly predicted by school type attended and did not predict medical undergraduate exam performance.

Analysis of data from the first medical and dental application cohort to use the UKCAT in 2006 has previously been conducted in order to explore to what extent A level attainment and UKCAT scores could be predicted from socioeconomic status [10]. A sub-sample of the 2006 application cohort where A level grades and UKCAT scores were available was used in this analysis (N = 9,884). As UKCAT and A level attainment scores were considered skewed these were treated in the analysis as dichotomous dependent variables. The authors observed that both A Level attainment and UKCAT performance were associated with a number of sociodemographic variables (sex, ethnicity, socioeconomic background and school type attended). The UKCAT test scores were observed to be significantly correlated with attainment at A level examinations (r = .39) [10]. The treatment of UKCAT scores and A level attainment as dichotomous outcomes might have led to informational loss and precluded a more detailed evaluation of the magnitude of the effects of the predictor variables on the outcomes under study. In addition, introduction of the A* grade at A level in 2010 may have produced a wider and more normal distribution of A level tariffs (as defined by the Universities and Colleges Admissions Service [UCAS] scoring system of school exam grades). Thus, the A* grade may permit greater discrimination between medical and dental applicants at the upper range of ability. Therefore it appeared timely to analyse data from a separate and more recent cohort of UKCAT candidates with two aims:

1. To evaluate to what extent the observations reported in the 2006 application cohort [10] are consistent in a subsequent sample of UKCAT candidates (i.e. evidence that the properties of the UKCAT are temporally stable).

2. To explore, in greater detail than was previously possible, the extent to which UKCAT scores are predicted by sociodemographic status in comparison with A level attainment. In particular, whether a candidate speaks English as a second language is now recorded within the UKCAT dataset. Thus the potential impact of this factor on both A level and UKCAT performance can be explored.

## Methods

### Data preparation- A level and UKCAT scores

For this analysis data relating to medical and dental applicants to the UKCAT consortia of universities in the 2009 round of applications (for 2010 entry) were used. Of the 23,719 individuals who sat the UKCAT in 2009 only 8,180 had complete data on the six sociodemographic predictor variables (ethnicity, age, school type attended, socioeconomic background, age at which English first spoken and sex). It was only information from these candidates with complete data that were included in the analysis. This approach, using ‘listwise deletion’, was taken so that the final sample investigated remained the same across all analyses.

In terms of managing the educational attainment data; A level grades in *general studies* and *critical thinking* were excluded, as were duplicated observations where the subject, grade and candidate unique identifier were identical (535 duplicate exam grades were deleted in the latter case). Examination outcome entries where the subject was the same but the grade differed for a candidate were assumed to be resits. In such cases the lowest grade was retained. This assumption was made for several reasons:- firstly, the dates of sitting of the examinations were unavailable; secondly, the first sitting was assumed to reflect a candidate’s academic potential more accurately than subsequent sittings; and thirdly, medical and dental schools often only accept grades at first sitting as meeting entry requirements. Consequently 810 presumed ‘resit’ exam grades were deleted. The UCAS tariff scores for a candidate’s best three A level grades were summed (that is, A* = 140, A = 120, B = 100, C = 80, D = 60 and E = 40 points). Thus the maximum summed tariff that a single candidate could obtain was 420 points (i.e. A*A*A* grades). Standardised z scores for both ‘best of three’ summed A level tariff and UKCAT total score were also derived (i.e. mean of zero and a standard deviation [SD] of one). This standardisation was intended to permit a certain amount of comparison between UKCAT and A level tariff scores. The distribution of standardised A level tariffs and UKCAT scores were examined graphically using histograms and quantile (Q-Q) plots to assess for degree of normality and to allow selection of appropriate estimation procedures. A Q-Q plot produces a graph of quantiles of the variable against quantiles of the normal distribution, allowing the visual identification of marked departures of a distribution from normality. This approach is recommended over simple reliance on significance tests for normality, such as the Kolmogorov-Smirnov test, which may be overly sensitive in certain circumstances [11].

### Data preparation- sociodemographic data

The dichotomisation of sociodemographic variables was guided by previous research on widening participation in medical and dental education [12] and informed by an initial univariate exploration of the dataset. For example, previous research has reported that students educated at state grammar schools (that usually include some element of criterion-based selection for admission, in contrast to other types of state school) do not have poorer performance on the UKCAT, at A level, or in university compared to those receiving an independent (private) schooling [10]. Thus, for the purpose of dichotomising candidates into those from WP categories it seemed reasonable to classify those from state grammar schools in the same group as those from an independent school background. This assumption was supported by an exploratory univariate analysis that did not uncover evidence of disadvantage for grammar school students compared to independently schooled individuals when applying to medical or dental school. Likewise, previous research demonstrated a disadvantage when applying for medical school for those reporting their ethnicity as anything other than ‘White’ [5]. Consequently ‘non-White’ was the WP category created for ethnicity. Some descriptive analysis was conducted using more broadly defined ethnic groups (‘White’, ‘Black’, ‘Chinese’, ‘Asian’, ‘Mixed’ and ‘Other’). However, the numbers in each of these latter ethnic categories was generally too small to adequately power more detailed modelling in relation to these groups. Regarding socioeconomic background, the UKCAT database records socioeconomic status using a simplified version of the socioeconomic classification system utilised by the National Office for Statistics, based on the National Statistics Socio-Economic Classification (NS-SEC) [13]. As in previous research on WP in medical and dental education we classified those who reported a NS-SEC rating of four or greater as being from a non-professional socioeconomic background. Thus this category was equivalent to the classifications of ‘*lower supervisory/technical*’ and ‘*semi-routine/routine*’. It should be noted that this method of WP group definition differs from the approach to categorising socio-economic status taken by James et al. [10]. In this latter study the authors used the *highest* (most advantaged) category of the simplified NS-SEC system, ‘*managerial/professional*’ as the socio-economic predictor variable. In contrast the present study used the two *lowest* parental occupation categories to represent relative socioeconomic disadvantage as a predictor of A level and UKCAT performance. We classified those learning English after the age of two years as having English as a second language (EASL).

### Data analysis

**Figure 1:**
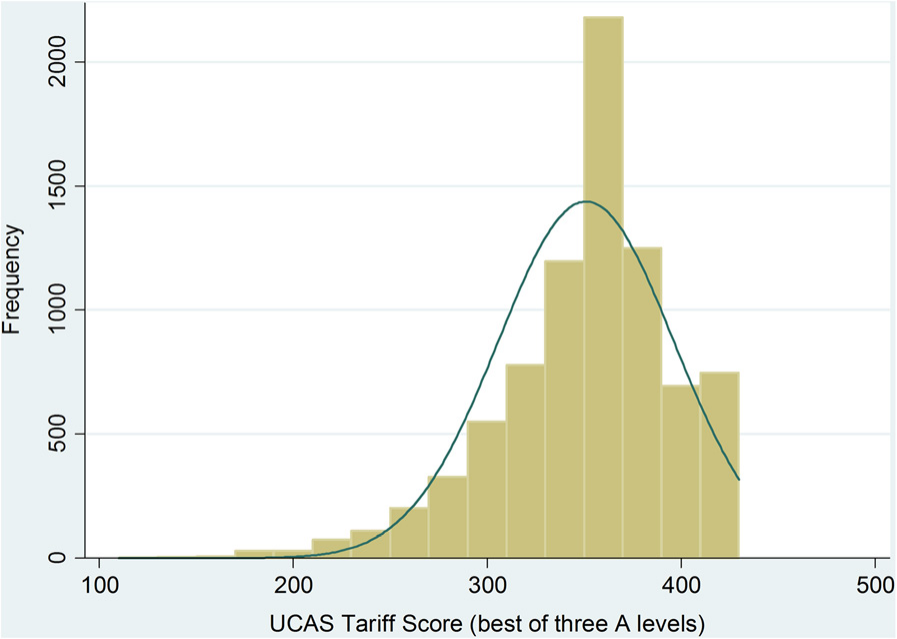
Histogram of the A level summed tariff scores ("best of three") for the sample (N = 8,180) with a normal distribution line superimposed.

**Figure 2:**
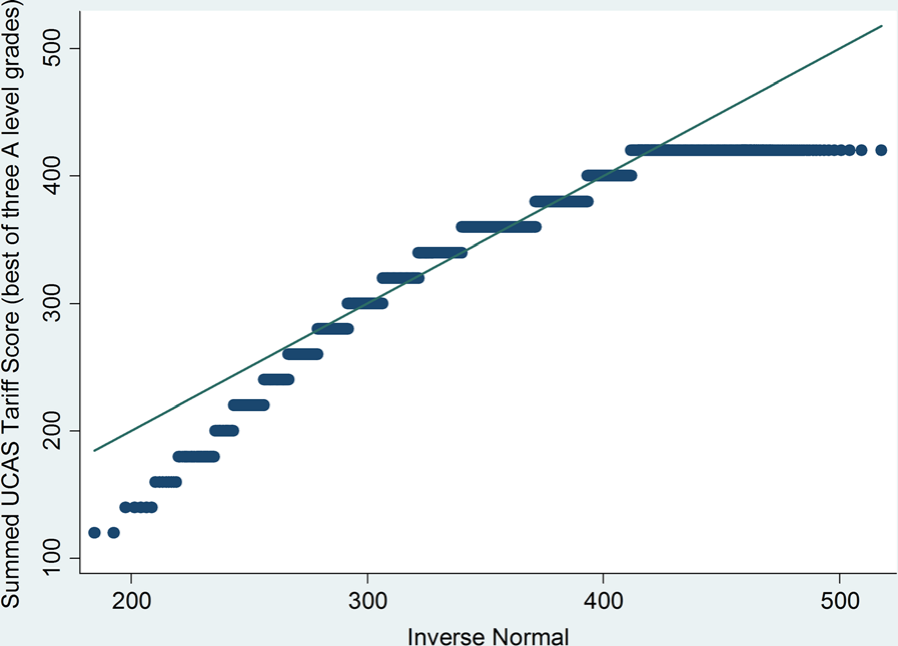
Q-Q plot of standardised A level tariff for best of three A levels for the sample (N = 8,180).

**Figure 3:**
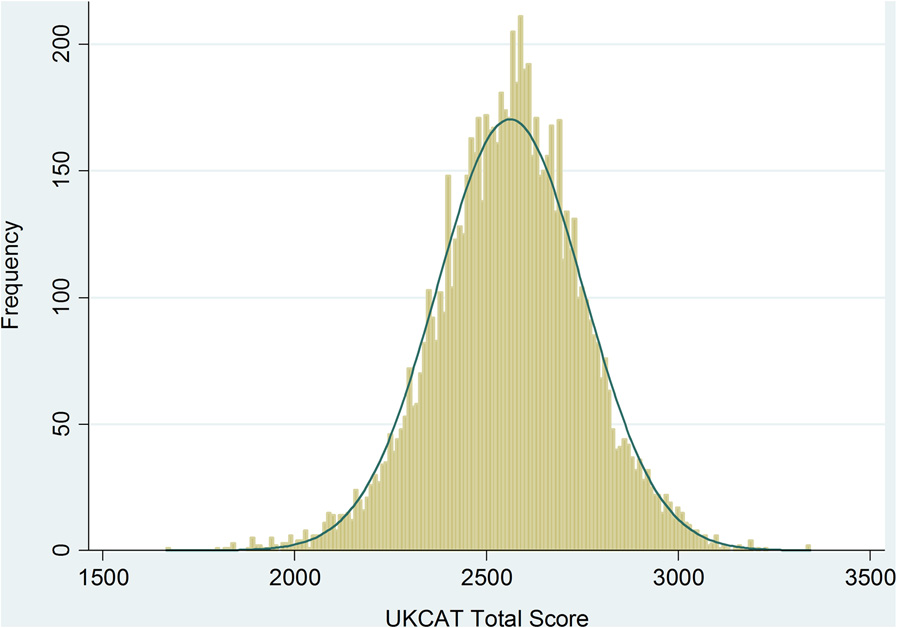
Histogram of the total standardised UKCAT scores for the sample (N = 8,180) with a normal distribution line superimposed.

**Figure 4:**
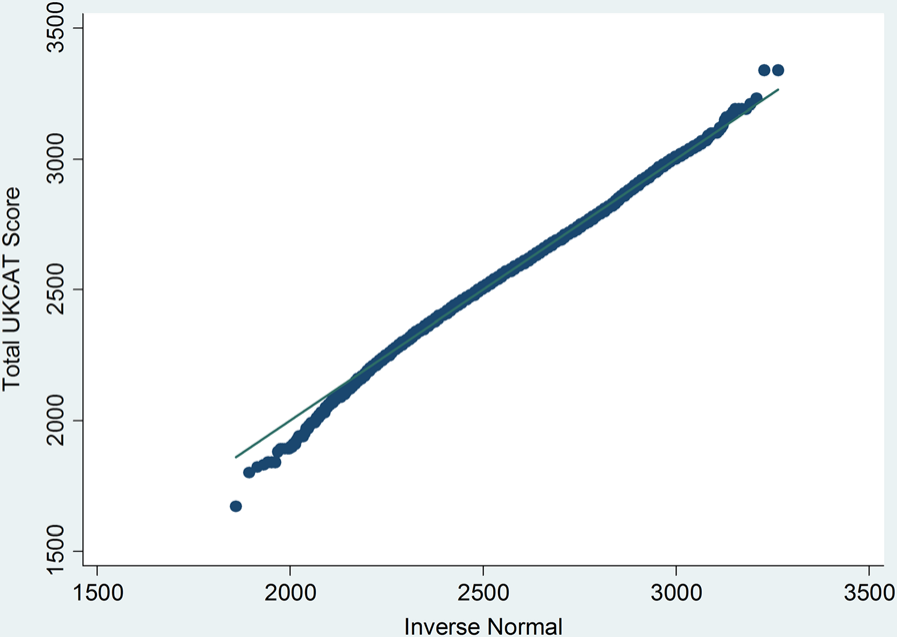
Quantile (Q-Q) plot of the UKCAT total score for the sample (N = 8,180).

We used Stata v12 MP for data analysis. The distribution of the "best of three" A level tariff scores appeared to be represented by a roughly normal distribution that was right censored at 420 points as only three grades had been retained (i.e. 3 A*s was the maximum possible attainment- see Figure 1). The Q-Q plot highlighted the censored nature of the tariff score and that those less than two standard deviations away from the mean departed from normality (Figure 2). It was thus considered appropriate to model the data using a Tobit regression that could accommodate the right-censored nature of the tariff score [14] with bootstrapped standard errors that would allow for the moderate departure from normality. On inspection, total UKCAT test scores were normally distributed (Figure 3) although a Q-Q plot highlighted some departure from normality at extreme low scores (Figure 4). However, this slight departure from normality did not appear to justify the use of non-parametric methods of deriving standard errors. Thus linear regression was employed to estimate the influences on UKCAT score. The association between WP group status and UKCAT subtest scores were compared using linear regression. The UKCAT subtest scores were also approximately normally distributed and, given the relatively large number of observations, linear regression was also utilised in their analysis. Thus Tobit and linear regressions were utilised, as appropriate, for both univariable and multivariable models. Scores were compared between the UKCAT and A level tariffs using a Spearman rank correlation (to allow for any non-normality at lower values).

Following univariate regression analyses, multivariable regression analyses were performed. Two sets of regression analyses were conducted using A level tariffs then UKCAT scores as the outcomes of interest, respectively. In both cases the WP sociodemographic categories served as the predictor variables. Only six predictor variables were used in the multiple regression models and the mean variance inflation factor for a model including all of these was relatively small at 1.09. This latter value implies that the standard error for the estimated coefficients would be, on average, increased by approximately 9% due to multicollinearity of the predictor variables. Consequently, rather than taking a step-wise approach to model building, all predictors were included in the multivariable regression models. Where A level summed tariff score was used as the dependent variable the model was estimated using a Tobit regression using bootstrapped derived standard errors. Thus two differing regression methods (Tobit versus linear regression) were used to produce regression coefficients, intercepts and associated 95% confidence intervals (via bootstrapping in the case of the Tobit regression). As the dependent variables used in the multivariable models were z scores the coefficients were produced in the metric of standard deviation units. This allowed, to some extent, an appreciation of the relative magnitude of ability of each sociodemographic category to predict A level achievement and UKCAT score. Nevertheless, it should be noted that the regression coefficients derived from a Tobit regression differ slightly in conceptual terms from those derived from a linear regression; Tobit coefficients are estimated taking into account the probability that a predictor is actually above the observable limit. It should also be noted that where standardised confidence intervals do not overlap there may be a true difference in the comparative sensitivity of the outcome metric (i.e. A level tariff vs UKCAT score) to the sociodemographic variable. Conversely, where there is a modest degree of overlap in confidence intervals one cannot assume that no difference exists at the 95% confidence level (though such a difference would not exist at the 99% confidence level). Thus, some additional caution must be exercised when attempting to make inferences by comparing the confidence intervals for the coefficients from these two different regression methods.

### Ethical approval

At UKCAT registration students were informed that the information would be used for educational research and evaluation of the UKCAT and that the results would be published in a form in which individual students could not be identified. Thus the data utilised was routinely contemporaneously collected and anonymised. Consequently, exemption from an external ethical review was confirmed in writing by the Chair of Durham University School for Health Research Ethics Committee.

## Results

### Study group

The socioeconomic characteristics of the study and non-selected groups are depicted in Table 1. Compared to the non-selected UKCAT candidates (n = 15,539) those in the selected sample were more likely to be under 21 years old at application, of White ethnicity, have attended an independent/grammar school, and speak English as a first language (all differences in proportions significant at the p < .001 level on Chi-squared testing).

**Table 1:** Socioeconomic characteristics of the study and non-selected groups

**Characteristic**	**Study group (n = 8,180)**	**Non-selected group (n = 15,539)**	***χ***^**2 **^**(p) for difference**
State schooled	4291/8180 (52.4)	4,609/8066 (57.1)	35.97 (<.001)
Non-white	3102/8180 (37.9)	6781/15140 (44.8)	102.55 (<.001)
Age > 20 years at application	61/8180 (0.7)	6563/15425 (42.5)	4600.00 (<.001)
Non-professional background	476/8180 (5.8)	505/8553 (5.9)	.06 (.8)
Male	3628/8180 (44.3)	6841/15539 (44.0)	.23 (.6)
EASL	1249/8180 (15.3)	4033/15374 (26.2)	368.92 (<.001)

Proportions of candidates with that characteristic are shown along with percentages and the chi-squared and associated p value for the inter-group differences.

### Univariate analyses

It should be noted, that when interpreting standardised scores and regression coefficients, for A level tariffs each standard deviation unit (SD) is equivalent to around 45 UCAS points. For the UKCAT scores each SD is worth around 200 points.

Using Spearman’s rho, total UKCAT score was observed to be correlated with A-level tariff (rho = .43, p < .0001). The UKCAT subtest scores significantly (all p < .0001) correlated with A level tariffs with rho values of .28 (verbal reasoning) to .36 (quantitative reasoning). The results of the univariate Tobit and linear regressions for the prediction of A level and UKCAT attainment respectively by WP status are depicted in Table 2. All WP variables were significantly negatively predictive of A level and UKCAT performance. However from Table 2 it can be seen that, in this sample, the effect of sex on A level and UKCAT performance was different; on average, males did not perform any better on A levels compared to females (coefficient .01of a SD; 95% confidence intervals; –.04 to .06; p = .6). In contrast, on average, males scored .17 of a SD higher than females on the UKCAT. The unadjusted effect of being from a non-professional socioeconomic background was comparable for both A levels and the UKCAT with those candidates scoring, on average .3 to .4 of a SD lower on both outcomes compared to those reporting being from NS-SEC classification 4 or 5. Being over 20 years old at application is also associated with lower unadjusted scores on both A level tariff and UKCAT (though it should be noted only 61 older individuals were included in the study sample). It can also be seen in Table 2 that the unadjusted coefficients for ethnicity and EASL status as predictors of UKCAT score are larger in magnitude than those for A levels, with non-overlapping 95% confidence intervals.

**Table 2:** Results of univariate regression analyses of standardised A level summed tariff and UKCAT total score

**Predictor variable**	**Outcome variable**	**Intercept (score)**	**Coefficient**	**p**^**§**^	**95% confidence intervals for regression coefficient**
State school^†^	UKCAT	.16	-.31	<0.001	-.35 to - .27
	A level	.21	-.40	<0.001	-.45 to - .36
Non-white ethnicity	UKCAT	.16	-.43	<0.001	-.47 to - .38
	A level	.11	-.29	<0.001	-.34 to - .25
>20 years at application	UKCAT	.01	-.68	<0.001	-.93 to - .43
	A level	.01	-.88	<0.001	-1.21 to - .55
Non-professional background	UKCAT	.02	-.41	<0.001	-.51 to - .32
	A level	.02	-.36	<0.001	-.45 to - .26
Male sex	UKCAT	-.08	.17	<0.001	.13 to .22
	A level	-.01	.01	0.62	-.04 to .06
EASL	UKCAT	.07	-.47	<0.001	-.53 to - .41
	A level	.03	-.20	<0.001	-.26 to - .14

The dependent variables (both standardised as z scores) are regressed on to the WP sociodemographic categories and sex, which serve as the predictor variables.

ATobit regression was used where A level attainment was the dependent variable whilst linear regression was used where UKCAT score was the outcome.

^§^p values must be interpreted cautiously where confidence intervals are derived by bootstrapping due to the possibility of asymmetric probability distributions.

^†^Excludes state grammar schools.

The ability of WP group status to predict UKCAT subtest scores was also tested using a series of linear regressions. The results are depicted in Table 3. As can be seen from Table 3 all sociodemographic categories significantly negatively predicted subtest scores on the UKCAT. The only exception to this was that male sex significantly positively predicted performance on all the UKCAT subtests with the exceptions of the *decision making* score (which was negatively predicted by male sex) and the *abstract reasoning* score, which was not significantly predicted by sex.

**Table 3:** Results of univariate linear regressions of UKCAT subtest scores on WP categories and sex

**Predictor variable**	**Outcome variable**	**Intercept**	**Coefficient**	**p**	**95% CI**
State school (excluding grammar)	Verbal	603	-12.0	<0.001	-15.18 to -8.91
	Abstract	632	-16.7	<0.001	-19.96 to -13.50
	Quantitative	668	-21.8	<0.001	-25.20 to -18.37
	Decision making	691	-9.1	<0.001	-10.90 to -7.39
Non-white ethnicity	Verbal	613	-43.1	<0.001	-46.22 to -40.01
	Abstract	625	-5.5	0.001	-8.88 to -2.20
	Quantitative	666	-24.3	<0.001	-27.84 to -20.82
	Decision making	689	-8.9	<0.001	-10.72 to -7.10
>20 years at application	Verbal	597	-49.4	<0.001	-67.61 to -31.11
	Abstract	623	-28.0	0.004	-46.88 to -9.17
	Quantitative	657	-38.3	<0.001	-58.28 to -18.31
	Decision making	686	-15.0	0.004	-25.27 to -4.73
Non-professional background	Verbal	599	-35.3	<0.001	-41.95 to -28.60
	Abstract	623	-12.9	<0.001	-19.87 to -6.02
	Quantitative	658	-20.7	<0.001	-28.06 to -13.38
	Decision making	686	-10.5	<0.001	-14.22 to -6.69
Male sex	Verbal	593	8.9	<0.001	5.74 to 12.06
	Abstract	622	.7	0.69	-2.60 to 3.93
	Quantitative	645	25.8	<0.001	22.38 to 29.22
	Decision making	687	-2.0	0.03	-3.78 to -.22
EASL	Verbal	604	-46.9	<0.001	-51.17 to -42.67
	Abstract	624	-7.5	0.001	-11.99 to -2.97
	Quantitative	660	-24.2	<0.001	-28.92 to -19.41
	Decision making	687	-11.4	<0.001	-13.81 to -8.92

Coefficients are provided in unstandardised form in the original metric of the UKCAT score (mean subscale score for this sample approximately 600, SD of 75).

Performance on the UKCAT and at A level was also analysed according to reported ethnicity. The results are depicted in Table 4 and demonstrate that, by and large, ethnic groups were ranked in the same way according to both average A level grades achieved and mean UKCAT summed score. The only exception to this was that applicants describing themselves as of Chinese ethnicity had a slightly higher mean A level tariff score compared to those reporting White ethnicity. However, the latter group had the highest average UKCAT score of any ethnicity.

**Table 4:** A level attainment and UKCAT performance according to the four main ethnic groups

**Reported ethnicity**	**Mean UKCAT score (SD)**	**Mean A level tariff (best of three (SD))**
White (N = 5078)	2592.9 (182.8)	356.1 (41.6)
Chinese (N = 256)	2566.4 (194.2)	366.3 (40.9)
Mixed (N = 265)	2561.4 (203.4)	350.3 (44.3)
Asian (N = 1997)	2513.9 (192.0)	342.7 (49.4)
Other (N = 232)	2496.4 (171.8)	342.3 (50.6)
Black (N = 352)	2426.0 (186.9)	320.6 (53.4)

A level attainment (best of three grades) and UKCAT performance (total score) according to the four main ethnic groups.

### Multivariable regression analyses

The results of the Tobit multivariable regression of A level tariff score on the WP predictors are depicted in Table 5. The results indicate that all the WP variables are significantly predictive of A level tariff score with the exception of sex and EASL (English as a second language). It should be noted that the pseudo R^2^ for the model was only .025, indicating that, despite having four statistically significant predictor variables included, the overall model predicted only a very small portion of the variance in the outcome variable.

**Table 5:** Results of a multivariable regression of A level tariff score on sociodemographic category of applicant

**WP predictor**	**Coefficient (standardised)**	**p**	**95% CI (standardised)**
State school	-.40	<.001	-.45 to - .36
Non-white	-.30	<.001	-.36 to - .24
>20 years at application	-.65	<.001	-.93 to - .37
Non-professional background	-.17	.004	-.28 to - .05
Male sex	.02	.42	-.03 to .07
EASL	.01	.81	-.07 to .10
Intercept	.33	<.001	.29 to .37

Results of a multivariable Tobit regression of A level tariff score on sociodemographic category of medical or dental applicant (N = 8180).

Regression coefficients and their 95% confidence intervals (derived via bootstrapping) are given as standardised scores (in SD units). Each unit is worth a tariff score of 45 points. Pseudo R^2=^.025, Log likelihood -11315.

The results of the multivariable linear regression of UKCAT total score on WP predictors are depicted in Table 6. The results indicate that all the WP variables are significantly predictive of UKCAT score at or below the p = .001 level. As with the multivariable model for A level tariff prediction, it should be noted that the R^2^ for the model was only .088, indicating that, even with six statistically significant predictor variables included, the overall model predicted only a small portion of the variance in the outcome variable.

**Table 6:** Significant, independent sociodemographic predictors of standardised UKCAT total score from a multivariable linear regression (N = 8,180)

**WP predictor**	**Coefficient (standardised)**	**p**	**95% CI (standardised)**
State school	-.31	<.001	-.36 to - .27
Non-white	-.36	<.001	-41 to - .31
>20 years at application	-.40	.001	-.65 to - .16
Non-professional background	-.17	.001	-.26 to - .08
Male sex	.18	<.001	.14 to .22
EASL	-.23	<.001	-.30 to - .17
Intercept	.27	<.001	.23 to .31

Regression coefficients and their 95% confidence intervals and are given in parenthesis as standardised scores (in SD units). Each unit is worth approximately 200 UKCAT points. Pseudo R^2^ = .088, MSE = .96.

## Discussion

In relation to the first aim of this study the findings suggest that the properties of the UKCAT are relatively temporally stable. As with the previous published analysis by James et al. [10] we confirmed a number of observations regarding the UKCAT scores in relation to other sociodemographic and educational variables:

1. That performance on the UKCAT and at A levels are moderately correlated.

2. That candidates from an independent or grammar school tend to achieve higher scores/grades at both the UKCAT and at A level compared to those who report a non-grammar school state education. This effect is apparent even after controlling for the effect of other predictor variables.

3. That candidates reporting themselves as of White ethnicity, on average, achieve higher A level tariffs and UKCAT scores than those describing themselves as Non-white. This effect is apparent even after controlling for the effect of other predictor variables.

4. Candidates from non-professional socioeconomic backgrounds were observed to achieve, on average, lower scores on both the UKCAT and at A level, even after controlling for the effects of other predictor variables.

5. That male sex independently and significantly predicted higher total UKCAT scores. This effect is also apparent for the *verbal reasoning* and *quantitative reasoning* UKCAT subtest scores.

When comparing the results from the present study and those reported by James et al. it should be borne in mind that our WP categories were coded in the reverse direction to the latter study [10]. However, as highlighted above, allowing for this difference the results of the two studies were largely consistent. Nevertheless a number of our observations were in contrast to the results reported by James et al. in the earlier cohort. Firstly, unlike the previous report, we did not observe that males performed significantly better at A level compared to females. Also, in the present study, males did not score significantly higher on *abstract reasoning* and *decision making* scales compared to females, conflicting somewhat with the findings of James et al. where *decision making* was the only subtest where a sex difference was not apparent. There are potential explanations for these apparent inconsistencies (see later).

Our second aim was to explore in further detail the sociodemographic predictors of UKCAT performance and contrast these with those observed for A level attainment. Certainly the use of UKCAT scores and A levels as continuous outcome measures has allowed for a more in-depth comparison of the two metrics of ability. However, in the event, there were only a relatively small number of additional conclusions we could draw from this more detailed approach, which also used EASL status and age as additional predictors, compared to the previous study in the 2006 cohort:

1. That UKCAT performance is independently predicted by both ethnicity and by EASL status; those individuals who report their ethnicity as ‘White’ and have English as a first language, on average, score more highly on the UKCAT than those reporting Non-white ethnicity and learning English after the age of two. In contrast, A level performance was only independently predicted by ethnicity, with those of White ethnicity achieving, on average, higher grades than those reporting ethnicity as non-White. This suggests that culture and language skills may have a somewhat larger negative impact on UKCAT compared to A level performance.

2. When raw data is analysed according to reported ethnic group (e.g. White, Asian, Black etc.) average performance at A level is generally ranked in the same way as that for UKCAT performance, with those reporting being of White/Chinese achieving the highest scores/grades and those reporting Black ethnicity the lowest. The difference between these highest ranking groups and the lowest is considerable, at roughly one standard deviation for both A level and UKCAT performance.

3. Whilst candidates from an independent or grammar school tend to achieve higher scores/grades at both the UKCAT and at A level compared to those who report a non-grammar school state education there is some suggestion from our results that this school-type bias may be more pronounced for A levels than for the UKCAT. For example, whilst the type of school attended is a significant univariate predictor of UKCAT score, the effect seems less pronounced than that for A levels; indeed the 95% confidence intervals touch but do not overlap (see Table 2). However, as highlighted earlier in the methods section, the regression coefficients derived from Tobit and linear regression may not be directly comparable and consequently some caution must be exercised in interpreting this finding.

4. Older candidates (those over 20 years at the time of application) were more likely to report, on average, poorer A level grades and lower UKCAT scores. However, as most older candidates (all except 61 individuals over 20 years) were excluded on the basis of missing A level data this observation should be treated cautiously.

5. That compared to females, males tend to perform less well on the *decision making* subtest of the UKCAT. No overall sex differences for the *abstract reasoning* subtest were observed in this analysis.

Thus we can conclude that some socioeconomic bias in the UKCAT scores exists but that this differs in a number of respects from that observed for A level attainment. Therefore, when considering issues relating to WP in medical and dental education the picture is more complex than simply favouring one metric of ability over another. Thus, these findings suggest that the UKCAT may be prone to more bias in some respects compared to A levels and less in others. Compared to A level performance the UKCAT may be more prone to effects related to sex. Moreover, whilst both metrics of ability show bias in favour of those reporting White ethnicity the UKCAT may be especially sensitive to linguistic ability, compared to A levels. The present sample largely took A levels in science and maths. These subjects may test language and communication skills less rigorously than the humanities. It is therefore unsurprising that the UKCAT appears to ‘penalise’ EASL status to a more significant degree compared to A levels in the present sample. In contrast, there were some suggestions from the data that UKCAT may, as a metric of ability, be less biased in favour of candidates from an independent or grammar school background than A level grades. Thus, the UKCAT may potentially offer some complimentary, if not incremental, value alongside educational attainment measures in relation to the medical and dental school selection process.

### Further comparison with previous findings

This study builds on the previous work investigating sociodemographic predictors of A level and UKCAT performance [10]. Our study sample was relatively comparable with the subgroup of UKCAT candidates providing data in this earlier study. The present sample used was slightly smaller in that we only included those with complete sociodemographic information, as opposed to just non-missing A level data. In practice this meant that it was mainly those under 21 years that lacked information on socioeconomic background that were excluded from the final sub-sample for analysis, as this was the principal WP variable missing, aside from A level attainment. It should be noted, however, that many older individuals would have already been excluded on the basis of missing A level data. Nevertheless, as in the present sample, the sub-group of 2006 candidates included in the previous study tended to be younger, more likely to be of White ethnicity have attended an independent/grammar school (EASL status was not available in the 2006 cohort). As outlined earlier, our findings were largely consistent with the observations reported in this earlier study. However, it is important to consider the apparent inconsistencies in the findings between these two studies. Firstly, our lack of any observed sex difference in A level achievement, in contrast to the findings of James et al. [10], can be explained by the differing ways that the metric of A level performance was constructed. In the present study we created a tariff score by summing the UCAS for the three best exam grades (excluding *general studies* and *critical thinking*) irrespective of whether the A levels were ‘pure’ science (i.e. chemistry, biology, physics and mathematics). Indeed, James et al. report only slightly higher average tariff scores for pure science subjects in males and no sex difference in overall average tariff scores. Thus, our observations are largely consistent with those reported by James et al., though we are unable to rule out the effects of a recognised recent secular trend towards males obtaining more top grades in science A levels compared to females [15]. Secondly, in the present study, males did not score significantly higher than females on the *abstract reasoning* and *decision making* scales. This appears to contrast somewhat with the findings of James et al. where *decision making* was the only subtest where a sex difference was not apparent. However, these inconsistencies may be relatively trivial once the differences in analysis approach are accounted for. Firstly, the males in our cohort performed more poorly than females on the *decision making* items, although the magnitude of this difference was slight and the p value for significant testing (p = .027) could be considered modest given the number of observations in the analysis. Thus, it may have been the case that by using a dichotomous outcome for UKCAT scores the James study may have been underpowered to detect a slight sex difference in performance on this subscale. Similarly, in the case of the *abstract reasoning* subtest this previous study reported a slight (but statistically significant) tendency for males to perform, more poorly on this element of the UKCAT; males had a 16% lower odds of scoring above the 30^th^ centile on this particular subtest. In contrast we observed no significant sex difference. However, we utilised UKCAT scores as a continuous, rather than as a dichotomous metric. Although these findings are not included in our results section, as with the report by James et al., we noted a slight excess of females scoring above the 30^th^ centile on *abstract reasoning* (1,397 females compared to 1,186 males). Thus, our results are largely consistent with those reported by the earlier study once the differences in methodologies are accounted for.

A previous study demonstrated that use of the UKCAT as a threshold score in the admissions process appears to ameliorate the disadvantage faced by lower socio-economic groups when applying to medical schools [5]. In addition, use of the UKCAT scores as a threshold in the admissions process was associated with increased odds of entrants being male, from a low socioeconomic status background and a state (non-grammar) school (the latter trend not reaching statistical significance). In contrast, universities placing less emphasis on use of the test were more likely to admit entrants with relatively low academic attainment and with English as a second language. These observations are generally consistent with the properties of the two performance metrics as reported in this present study. Thus, the present findings imply that it is mainly the differences in sensitivity to sociodemographic factors (i.e. bias) between A levels and the UKCAT that are driving these differences. The obvious exception to this is that in the present study we found no evidence that the UKCAT was less biased than A levels against those from a non-professional socioeconomic background than were A levels. However, it is possible that if UKCAT performance is less sensitive to schooling than A level attainment then this difference may be at least partly mediating this previous observation [5]. Moreover, our present results do not explain why universities that place little emphasis on the test scores may be more likely to have entrants with below average educational performance.

### Limitations

The primary limitation of this study was that analysis could only be conducted on a minority of applicants for 2009, due to missing data. This limits our confidence in the generalisability of these findings to the wider population of UKCAT candidates. In particular, the individuals with complete data were more likely to be of White ethnicity, have attended an independent/grammar school, be younger and to speak English as a first language. Thus, we must be extremely cautious in drawing any conclusions about the association between WP variables and UKCAT performance in sub-groups of candidates who belong to the opposite sociodemographic categories. Missing data modelling in conjunction with imputational approaches could have been used to inform sensitivity analysis (i.e. assess how strong the findings are under different assumptions). Such an approach has been previously employed with educational data to provide an indication of the extent to which data are missing at random as opposed to being non-ignorable [5]. However, it was felt that imputational approaches could have added a significant degree of uncertainty to the dataset, especially as more than one variable would have had to be imputed. In addition the final sample providing data for analysis would have been difficult to compare with the previous sub-group of candidates studied [10]. Inclusion of advanced qualifications other than A levels may have modestly addressed the missingness but also potentially added a degree of complexity and possible confounding; it is uncertain to what extent other tests of educational attainment are equivalent to each other (e.g. Scottish Highers vs A levels). Thus, on balance, it was felt that restricting the analysis to those with complete data would enhance the internal validity of the findings, accepting that this would be at the expense of generalisablity of the results observed.

Whilst some descriptive analysis was conducted using more broadly defined ethnic groups it would have been desirable to have detailed modelling in relation to ethnicity. Certain ethnic groups (e.g. those describing themselves as ‘Black’) are relatively under-represented in UK medical and dental education whilst others are over-represented (e.g. Asians) in relation to the national population demographics [12].

A further limitation, as stated earlier, is that the use of Tobit regression, whilst necessary with censored data, still leads to some informational loss compared to linear regression, and the coefficients and confidence intervals produced by the two approaches (i.e. Tobit and linear regression) may not be easily comparable.

### Implications for practice and directions for future research

The UKCAT is a high stakes test; the psychometric properties of the test, in conjunction with a widespread adoption as part of the admissions process could at least partly determine the nature of the UK’s future medical and dental workforce. Both the present and a previous study report evidence of a certain degree of sociodemographic bias in the UKCAT responses. Firstly, possibly the most consistently reported of these is the observation that males achieve higher scores on the UKCAT than females. In the UK females are currently disproportionately represented amongst medical and dental school entrants. This issue has, at times, stirred up controversial debate [16]. Currently female doctors are more likely to work part-time [17] and retire early [18] compared to their male counterparts. Nevertheless, it should be highlighted that a previous study reported that female doctors were at lower risk of professional misconduct after qualification, even after adjusting for a number of potential confounding factors [19]. Moreover, in the UK, women may outperform men in certain medical undergraduate [20] and post-graduate exams [21]. This study has highlighted some differences in the differential sensitivities of the UKCAT subtests to sex. Thus our findings may assist universities making informed decisions about how much weight to place on each element of the UKCAT when selecting entrants.

The potential insensitivity of the UKCAT to educational background is certainly a factor that could help address the issue of widening participation in the professions. However, it should be highlighted that the WP agenda is not purely focused on issues of social equity and fairness; there is evidence from North American research that students drawn from minority populations may be more likely to eventually practice in areas that have been traditionally underserved by health care provision [22]. In the US attempts to address racial imbalances within the professions, including medicine, via the ‘affirmative action’ approach have proved controversial and have been the subject of a series of Court cases [23]. Earlier North American researchers have suggested that the use of cognitively based aptitude tests (such as the UKCAT) will never address the under representation of racial minorities in medical education as such instruments tend to produce similar mean raw scores according to ethnicity [24]. Rather, it has been postulated that the most plausible way of achieving a medical school population with a similar ethnic profile to the population from which they are drawn is to have quotas for each group. It has been suggested that these quotas can be fulfilled without any appreciable lowering of average academic performance at medical school [25]. In the UK such affirmative action-style approaches have not been adopted and even their legality would have to be tested.

Our findings suggest that the UKCAT test that may penalise those who do not speak English as a first language more severely than science-based A levels do. Nevertheless, fluency in spoken English has been reported to correlate significantly with patient and examiner ratings of global communication, which is considered a key attribute of a clinician [26]. It could therefore be argued that it is reasonable for the UKCAT to evaluate elements of linguistic ability such as verbal reasoning.

Future research should focus on obtaining further evidence regarding whether or not the UKCAT has the ability to predict undergraduate and post-graduate performance and progression, over and above that possible via traditional measures of educational attainment. Moreover, it may be that the content and delivery of the test can be modified to further decrease the sensitivity to educational background. In the field of education there is some evidence that ‘dynamic testing’ may be better at predicting an individual’s academic and potential compared to traditional (‘static’) cognitive assessments. This may be especially true where a candidate’s education has been poor or disrupted [27]. Dynamic tests create a learning environment within the test structure by providing novel situations and then evaluate the nature and number of prompts, hints and clues the candidate requires in order to achieve a correct response. Such tests correlate highly with traditional ‘intelligence tests’ but provide additional information relating to cognitive flexibility and learning potential [28]; attributes obviously pertinent to medical or dental practitioners.

## Conclusions

Both the UKCAT scores and A level performance are independently predicted by a number of sociodemographic group variables. The nature, and perhaps the degree, of these relationships differ to some extent, suggesting some incremental value in using the UKCAT scores to complement actual or predicted school achievement during the selection. These differences should be considered when designing medical and dental school admissions policy. In particular, universities need to consider how use of the test may impact on the proportions of course entrants who are male, from a state school background or who may have EASL.

## Competing interests

SN is Chair of the UKCAT Board. JMcL is a UKCAT board member. PAT is member of the UKCAT Research Panel. SN, JMcL and PAT are active members of the UKCAT Consortium and consequently all received reimbursement for expenses incurred as part of their work in connection with UKCAT consortium activity from the UKCAT Board. PAT and JMcl have received funding (following a competitive tendering process) from the UKCAT for conducting research on behalf of the UKCAT Board. The UKCAT Board encourages publication of the findings from well conducted research that contribute to a better understanding of how aptitude tests may contribute to undergraduate selection and medical and dental education as a whole. LW has no competing interests.

## Authors’ contributions

PAT lead on conception, design, statistical analysis and interpretation of data and is the guarantor of the paper. JMcL contributed to drafting, revising the article and critically appraising the content. LW contributed to the statistical analysis and drafting of the final article manuscript. SN contributed to revising the article and critically appraising the content. All authors (PAT, JMcL and SN) have approved the final version of the article submitted.

## Pre-publication history

The pre-publication history for this paper can be accessed here:

http://www.biomedcentral.com/1472-6920/14/7/prepub
